# Urban Form, Air Quality, and Cardiorespiratory Mortality: A Path Analysis

**DOI:** 10.3390/ijerph17041202

**Published:** 2020-02-13

**Authors:** Chaosu Li, Yan Song, Li Tian, Wei Ouyang

**Affiliations:** 1 Faculty of Innovation and Design, City University of Macau, Macau, China; chaosuli@live.unc.edu; 2 Department of City Planning, Shenzhen University, Shenzhen 518060, China; 3 Department of City and Regional Planning, University of North Carolina at Chapel Hill, Chapel Hill, NC 27599, USA; 4 School of Architecture, Tsinghua University, Beijing 100084, China; litian262@mail.tsinghua.edu.cn; 5 School of Public Administration, Renmin University of China, Beijing 100872, China; ouyangwei@ruc.edu.cn

**Keywords:** environmental health, PM_2.5_, urban form, cardiorespiratory mortality, China

## Abstract

With the unprecedented urbanization during the past three decades, air quality in many Chinese cities has been a serious issue which poses great challenges for urban sustainability. This study examines the health consequences of development patterns in China by establishing the linkage between urban form, air pollution level, and cardiorespiratory mortality rate. We assembled a dataset by compiling a series of variables from multiple sources, including China’s Disease Surveillance Points (DSP) system, which forms a nationally representative sample of mortality for the year 2005, Chinese census, satellite imagery, and the Chinese National Land Use Database. After controlling for local climate, demography, socioeconomics, and other pollution factors, this study finds that urban form elements (e.g., urban density, fragmentation level, forest/green space ratio) have significant influences on PM_2.5_ (atmospheric particulate matter with a diameter of less than 2.5 micrometers) concentration, thus influencing the incidence of cardiorespiratory mortality at the county level. These results may help explain how the type and pattern of development shape public health by influencing air quality and form an evidence-based land use policy to improve environmental quality and public health.

## 1. Introduction

During recent decades, with the unprecedented urbanization, rapid economic growth, and increased usage of automobiles, air quality in many Chinese cities has been extremely poor and has become an issue associated with increasing social unrest [1,2,3]. Ambient concentrations of fine particular matter in Chinese cities are much higher than the national standard and bring about serious environmental issues [2,4]. Major cities in China, such as Beijing, Tianjin, Nanjing, Zhengzhou, and Jinan, frequently suffer from urban smog, with the number of days of smog pollution exceeding 130 days a year [4]. The Health Effects Institute also suggests that Chinese cities face the worst air quality across different cities around world based on an extensive research of 175 countries [5].

In effect, excessive exposure to air pollutants can lead to several negative physical health outcomes, such as cardiorespiratory diseases, lung cancer, and stroke [6,7,8,9]. In the context of China, it is also worthwhile to note cardiorespiratory diseases and lung cancer have been recorded as the leading cause of mortality in the recent decade [10]. More recently, additional empirical evidence has been accumulated in the literature that exposure to air pollutants can cause mental health issues [11,12,13]. The health effects of air pollution and its appropriate regulation continue to be extremely important in China. According to recent studies, a 10 μg/m^3^ increase in airborne PM_10_ (atmospheric particulate matter with a diameter of less than 10 micrometers), could possibly reduce a Chinese resident’ life expectancy by 0.64 years, which is equivalent to 0.89 billion life-years based on China’s total population [3].

Meanwhile, there have been clear connections between urban spatial structure and air pollution. Relevant urban form policies have the potential to improve urban energy efficiency [14,15], carbon emissions [16,17,18], and human exposure to air pollution [19,20,21,22,23,24]. It is also worthwhile to note that relevant urban form strategies to mitigate air pollution are relevant not only because of their clear impacts on air pollutants, but also due to the potential co-benefit to combat climate change [2,3].

Currently, cities are increasingly paying more attention to urban form-related planning interventions that may shape urban air quality and public health [25,26,27]. Although existing studies have revealed the apparent relationship between urban form and air quality, the more comprehensive relationships among urban form, air quality, and health outcomes are not well established. In this regard, we aim to investigate the linkage between urban form, air pollution level, and public health in a more comprehensive manner and to further quantify the indirect effects of urban form on health outcome through air quality. We conduct this research in the context of China, which is of tremendous policy relevance, because the results are informative for people currently living in China and other people who are exposed to air pollution in developing countries. We first review current literature on urban form factors affecting air quality, as well as health outcomes associated with air pollution to establish the conceptual framework for this study. We present our data collection and coding, urban form measures, and the path model. We then present the results for a sample of 158 counties in China and set these in the context of existing literature. Additionally, we discuss the limitations of this study. Finally, we conclude with potential policy implications.

## 2. Prior Research

Connections between urban form and air quality are well established, especially in the recent literature [27]. Both simulation and empirical studies demonstrate the associations between various urban form measures and local air quality. Simulation studies usually explore different land use scenarios and support the notion that compact development improves urban air quality [28,29]. On the other hand, empirical studies in this field generally agree that urban form and land use features have modest but important impacts on urban and regional air quality [22,24,30,31,32,33,34]. Urban size, density, shape and contiguity (irregularity), fragmentation of urban patches, and urban forest amount and mixing level have been identified as valid urban form dimensions that can potentially capture these effects [22,24,30,31,32,33,34,35,36,37].

There has been a high degree of consensus among empirical studies that urban sprawl and fragmentary urban land use increase emissions of CO, NO_x_, PM_2.5_, and PM_10_ since dispersed and fragmented development could bring about a higher ratio of automobile use, longer trip length, and thus, worsen air quality [22,24,31,32,33,37,38]. Additionally, forest coverage level and urban-forest mixing level are significantly associated with air quality, which is evident in the literature since forests could potentially improve air quality conditions in urban areas [30,32]. Nevertheless, there are still inconsistent results within empirical studies. For example, the direction and magnitude of population density are currently disputed in the literature: Stone (2008) and Bereitschaft and Debbage (2013) suggest that higher population density is associated with lower air pollutant concentration (e.g., PM_2.5_ and O_3_). In contrast, Clark et al. (2011) find that higher population density is positively associated with population-weighed PM_2.5_ concentrations, and Rodríguez, et al. (2016) suggest that denser cities often times suffer from higher SO_2_ concentrations. More recently, Han et al. (2019) find that population density is related to PM_2.5_ concentrations, indicating pollution centralization and transportation congestion effects might be larger than effects of mode-shifting associated with density, especially in high-density urban areas [39]. Additionally, different research findings of urban shape complexity are reported by different empirical studies. Bereitschaft and Debbage (2013) suggest that a higher porosity level of urban areas may lead to increased duration of vehicle travel and its associated emissions, thus increasing air pollution levels. However, other studies do not find such significant associations [24,38].

Meanwhile, most existing research exploring the linkage between air pollution and health focus on health effects of air pollutant concentration on physical health. The majority of studies use cardiovascular or respiratory disease as a valid measure of the physical health outcome associated with air pollution. Recent studies also reveal that land use and urban form features may also affect mental health [12,40,41]. Although the empirical evidence on the impact of urban form measures on mental health is accumulating [40], the measurement of mental health is often times obscure, thus making it difficult to reveal any causal relationships. In summary, various physical and mental health outcomes of air pollution, especially physical health outcomes such as mortality and cardiorespiratory disease, have been well documented in prior studies.

It is worthwhile to note that the negative effects of air pollutant concentration on physical health have been well documented in the recent literature, especially in urban China [42,43,44,45,46,47]. Additionally, the existing literature generally agrees that cardiorespiratory causes of death are a valid measure of health outcome that have direct linkage to air quality. This study attempts to further advance the empirical understanding of how urban form shapes cardiorespiratory causes of death by influencing air quality. We attempt to explain air quality variation using urban form measures that have been well documented in the existing literature and single out the pathway of urban form measures in influencing cardiorespiratory causes of death, thus quantifying the indirect effect of each urban form measure.

## 3. Data and Methods

### 3.1. Mortality Data

**Figure 1:**
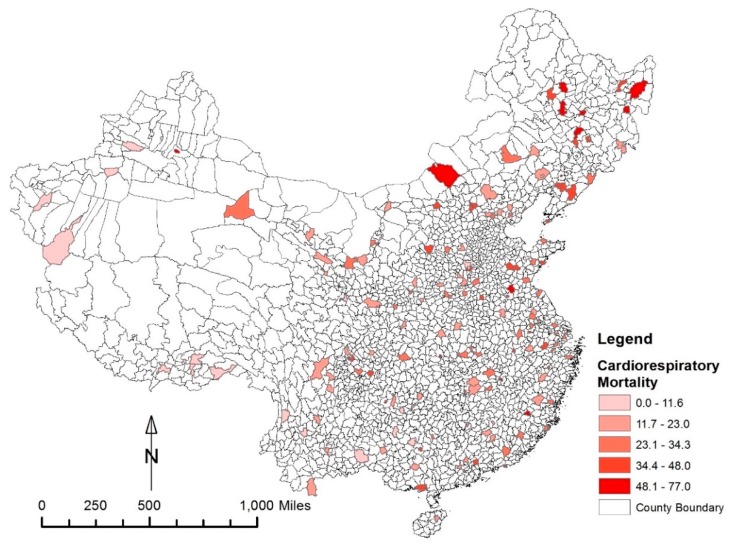
Spatial distribution of cardiorespiratory mortality rate (incidences/100,000 population) of Disease Surveillance Points (DSP) sample sites in the year 2005.

Our sample of mortality is taken from the China’s Disease Surveillance Points (DSP) system, which is a high-quality nationally survey conducted by the Chinese Center for Disease Control and Prevention; it contains detailed cause of death data verified by verbal autopsy (The verbal autopsy is a method of gathering health information about a deceased individual to determine his or her cause of death. Health information and a description of events prior to death are acquired from conversations or interviews with a person or persons familiar with the deceased and analyzed by health professionals or computer algorithms to assign a probable cause of death.) for a nationally representative sample of mortality of 158 counties in China in the year 2005. It is worthwhile to note that the year 2005 is the most recent year with available mortality data from the DSP system as well as nationwide PM_2.5_ data and the Chinese National Land Use Database all available. In this study, we use the death category of cardiorespiratory causes of death, which have been documented by previous literature as being directly associated with air pollution [2,3]. According to International Classification of Disease Revision 9 (ICD-9) used in this study, cardiorespiratory causes of death are lung cancer, heart diseases, vascular disease, and respiratory diseases. Figure 1 shows the spatial distribution of cardiorespiratory mortality rate (incidences/100,000 population) of DSP sample sites in the year 2005, which is the variable we use later in our path model.

### 3.2. Urban Form Measures

Based on previous studies in this field [22,23,24,31,32,33,34,48,49], four measures were used to evaluate urban form in various dimensions: urban density, fragmentation level, shape complexity, and forest/green space amount.

As is identified in the previous section, urban density has been regarded as a basic urban form measure that influences local and regional air quality [22,24,38,49]. In this study, population density is calculated based on gridded geographic datasets of the population in China obtained from the Institute of Geographic Sciences and Natural Resources Research, Chinese Academy of Sciences. The population density of each county is calculated by extracting the raster cells with population larger than zero and getting the total population of each county divided by the total area of the extracted cells. This measure is more precise than the traditional population density data in China because it excludes the areas without population.

As is also indicated in the previous literature, the fragmentation or aggregation levels of urban landscape are significantly associated with air pollution concentration [22,24,31,32,33,38,50]. Thus in this study, we include the urban cohesion index, which measures the aggregation level of the urban land use, since there has been accumulated evidence showing the linkage between urban land aggregation and air quality [33]. Urban cohesion index is calculated as follows [51,52]:

(1) $$\mathrm{CH}=\left(1-\frac{\sum_{j=1}^{n}Pij}{\sum_{j=1}^{n}Pij\sqrt{Aij}}\right)\times {\left(1-\frac{1}{\sqrt{N}}\right)}^{-1}\times 100$$

where CH is the urban cohesion index, *Pij* is the perimeter of urban patch *ij*, *Aij* is the total area of urban patch *ij*, *N* is the total number of cells in the urban land use. In this study, we extract the urban patches from the National Land Use/Cover Database of China in the year 2005 (30 m resolution) and use Fragstats 4.2 to calculate the urban cohesion index for each county.

Some empirical studies also identified that urban shape complexity has the potential to increase air pollution levels, since it may increase the duration of vehicle travel and its associated emissions [22]. The variable that measures the complexity of urban landscape used in this study is the commonly-used area-weighted perimeter-area ratio, with higher values indicating higher levels of urban boundary complexity [24,53]. The index is calculated as follows:

(2) $$\mathrm{PA}=\overset{n}{\underset{j=1}{\sum }}[Xij\times \left(\frac{Aij}{\sum_{j=1}^{n}Aij}\right)]$$

where PA is the area-weighted perimeter–area ratio, *Aij* is the total area of urban patch *ij*, *Xij* is the perimeter of the urban patch *ij* divided by the area of the urban patch *ij*. Again, we extracted the urban patches from National Land Use/Cover Database of China and used Fragstats 4.2 software to calculate this index for each county.

The last urban form feature that has important impacts on regional air quality in prior literature is forest amount and mixing level [32]. The relevant urban form variable we used in this study is forest/green space ratio, which is calculated as forest/green space area divided by total area for each county from the National Land Use/Cover Database of China. We also calculated the forest mixing level following McCarty and Kaza [32]. Since it is highly correlated with the dummy variable which indicates if the county is urban or not, we did not include the forest mixing level variable in our final model.

### 3.3. Conceptual Framework

**Figure 2:**
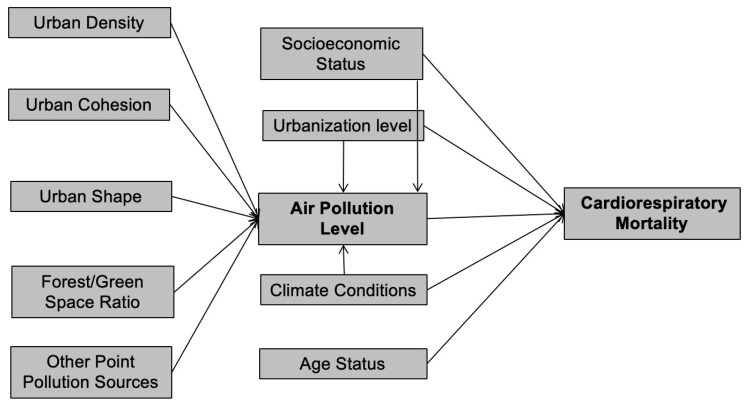
Conceptual framework and key relationships among urban form, air pollution level, and cardiorespiratory mortality.

The conceptual framework of this study, shown in Figure 2, summarizes the main relationship between urban form elements, air quality, and cardiorespiratory causes of death. The expected causal relationships begin with exogenous factors. Air pollution level and cardiorespiratory causes of mortality are two endogenous factors. Urban form elements (density, fragmentation, and shape), as well as forest/green space ratio, affect air pollution, after controlling pollution from other point sources, urbanization level, socioeconomic status, and climate factors. Further, the air pollution level influences cardiorespiratory causes of death after controlling for socioeconomic factors, urbanization level, climate conditions, and the residents’ age status. As stated previously, the key linkages we are interested in are urban form’s potential indirect connections to cardiorespiratory causes of death through air quality. It is necessary to note that there are potential interactions in this framework which are not captured. For example, it is possible that several variables for urban form also influence exposure of people to air pollution as well as potentially influencing air pollution concentrations themselves. We leave this for future exploration.

### 3.4. Variable Coding and Descriptive Statistics

In addition to urban form variables, a dataset from multiple sources was assembled at the county level for heating degree days (Heating degree day (HDD) is a measurement designed to quantify the demand for home heating. Higher values of HDD indicate greater needs for home heating in winter months. HDD has been widely used in energy studies and has been identified in previous studies as one of the variables associated with poor air quality in Chinese cities in winter.), urban county, GDP per capita, percentage of elderly residents, and pollution company density, to account for other factors that influence PM_2.5_ concentration level, and cardiorespiratory mortality, which have been identified in the previous literature and the conceptual framework (Table 1). Population-weighted concentrations of PM_2.5_ were used as the measure to evaluate air quality for each county, which were calculated using Global Annual PM_2.5_ Grids (http://sedac.ciesin.columbia.edu/data/set/sdei-global-annual-gwr-pm2-5-modis-misr-seawifs-aod/data-download4) in the year 2005 and 1 km Grid Population Dataset of China (http://www.geodoi.ac.cn/WebEn/doi.aspx?doi=10.3974/geodb.2014.01.06.v1).

Table 2 presents descriptive statistics for the measures used in the analysis. Unsurprisingly, the urban form measures and the cardiorespiratory mortality rate are skewed. Therefore, we should consider the non-normality-induced bias in the model selection.

### 3.5. Model Specification

To answer the research questions of this study, we developed a path model to account for the complexity of indirect relationships among urban form measures, air quality, and cardiorespiratory mortality, which are frequently used to model relationships in a complex system [54]. Path analysis was ideal for this analysis of urban form and cardiorespiratory mortality, since we intended to explore how urban form elements such as density, fragmentation, perimeter to area ratio, and green space, affect cardiorespiratory mortality ratio indirectly by influencing air quality.

Our path model was built based on the hypothesized linkages and conceptual framework discussed in the previous sections. Since a few of the variables in this study, especially the cardiorespiratory mortality rate, were heavily skewed, we employed the robust maximum likelihood (RML) estimation method, which corrects for non-normality-induced bias in the standard errors [55,56]. We specified urban form elements, pollution company density, percentage of elderly residents, GDP per capita, urban county, and heating degree days as exogenous variables. It should be noted that some of the exogenous variables might be correlated. Thus, we have calculated the Pearson Correlations between exogenous variables used. The results indicate that no correlation between exogeneous variables is high enough to create an instability in the parameter estimates of the path analysis: for example, the correlation between Heating Degree Days and Forest/Green Space Ratio is weak (Pearson Correlation Coefficient = −0.229). Similarly, the correlation between GDP per Capita and Percentage of Elderly Residents is not strong (Pearson Correlation Coefficient = 0.206). Urban County and Percentage of Elderly Residents also presents a weak correlation (Pearson Correlation Coefficient = 0.286). Annual PM_2.5_ level and cardiorespiratory mortality rate were endogenous variables. The following equations were estimated in STATA software:

CardioR_i_ = β_gsi_GDP+ β_usi_UrbanC + β_psi_PM + β_hsi_HDD + β_esi_EldR + ε_is_ (3)

PM_i_ = β_gti_GDP + β_pti_PopD + β_cti_CohenI + β_rti_PerimeR + β_fti_ForestR + β_dti_PollD + β_uti_UrbanC + β_hti_HDD + ε_it_ (4)

where:

CardioR = Cardiorespiratory mortality ratio within the county
GDP = Gross domestic product per capita of the county
UrbanC = Dummy variable indicating if the county is an urban county or not
PM = Population-weighted annual average PM_2.5_ within the county
HDD = Annual heating degree days of the nearest temperature station
EldR = Percentage of elderly residents within the county
PopD = Population density within the urban area of the county
CohenI = Urban Cohesion Index
PerimeR = Perimeter-Area Ratio
ForestR = Total forest/green space divided by total area within the county boundary
PollD = Total number of pollution companies divided by total area within the county boundary
ε = error coefficients
β = robust maximum likelihood estimates of independent variables.

## 4. Findings

**Figure 3:**
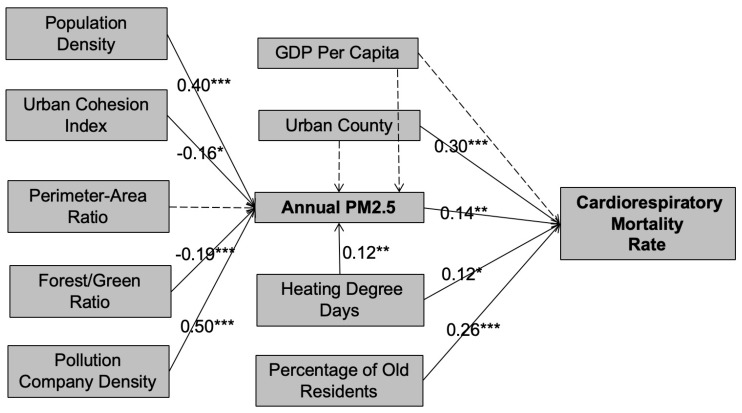
The estimated weights for the path model. Note: Standardized coefficient; *** denotes P < 0.01; ** P < 0.05; * P < 0.1. GDP: Gross Domestic Product.

The estimates of both coefficients (β) and standardized coefficients (St. β) of our path model are provided in Table 3. These estimates indicate that air quality, as measured by PM_2.5_ concentration, is influenced significantly by urban form elements, pollution company density, and heating degree days (Figure 3). Our results indicate that higher population density contributes to PM_2.5_ concentration at the county level. As shown in Table 3, a 10% increase in urban population density is associated with 4.0% increase in PM_2.5_ concentration level. The results also suggest that there exist negative direct effects of urban cohesion index and forest/green ratio on PM_2.5_ concentrations at the county level. Our model (Table 3) suggests that a standardized unit increase in urban cohesion index and forest/green space ratio would reduce the PM_2.5_ concentration level by 0.16 and 0.19 of a standardized unit, respectively. Nevertheless, our results do not reveal significant direct effects of perimeter-to-area ratio and PM_2.5_ concentration. As expected, pollution company density and heating degree days are predominant factors that explains the variation of PM_2.5_ concentrations at the county level: a standardized increase pollution company density and heating degree days would increase the PM_2.5_ concentration directly by 0.45 and 0.12 of a standardized unit, respectively.

It is also worthwhile to note that PM_2.5_ concentration significantly affects the cardiorespiratory mortality ratio at the county level. As is shown in our model, a 10% increase in PM_2.5_ concentration is associated with 1.4% increase in the cardiorespiratory mortality ratio at the county level. Similarly, as expected, the percentage of households with no tap water access, heating degree days, and urban county all have positive direct impact on cardiorespiratory mortality ratio at the county level.

The goodness-of-fit indices that are commonly used for the path model are appropriate in this analysis: the coefficient of determination (CD) is high (0.91), the root mean square error of approximations (RMSEA) is low (0.09), and the comparative fit index (CFI) and is high (0.89). These indices together suggest acceptable model fit.

**Figure 4:**
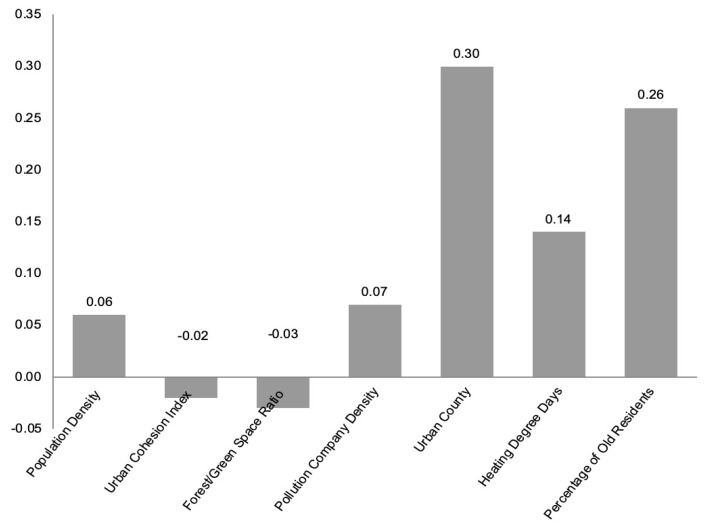
Standardized effects of urban form and other factors on cardiorespiratory mortality rate.

The total impact of the path variables on cardiorespiratory mortality ratio is presented in Figure 4. It is worthwhile to note that the largest standardized coefficient influencing cardiorespiratory mortality ratio is urban county (0.30), followed by percentage of elderly residents (0.26), Heating Degree Days (0.14), and pollution company density (0.07). The three variables of special concern in this study, urban population density, urban cohesion index, and forest/green space ratio, also impart significant indirect impacts on cardiorespiratory mortality ratio at the county level. That is, increased urban cohesion and more forest/green space are associated with lower cardiorespiratory mortality incidence. In contrast, increased urban population density is associated with higher cardiorespiratory mortality incidence at the county level. In comparison with other variables, the impacts of urban form elements on cardiorespiratory mortality are small (−0.02 to 0.06) and indirect.

## 5. Discussions

The empirical evidence from this study suggests that (1) forest/green space ratio within the county boundary is associated with lower PM_2.5_ concentration and thus lower cardiorespiratory mortality ratio; (2) urban cohesion level is associated with lower PM_2.5_ concentration and thereby lower cardiorespiratory mortality ratio within the county indirectly; and (3) increasing urban population density is associated with higher PM_2.5_ concentration.

The first two conclusions from this study are supported by existing literature, while the third one is not. The first conclusion confirms a large body of literature that has already shown green space effects to decrease air pollutant concentration [22,49]. The second conclusion also confirms a number of studies indicating that urban fragmentation is positively associated with CO, NO_x_, and PM_2.5_ emissions [24,31,32,34,38,49]. The third conclusion might be controversial compared with findings from previous studies. Regardless, we can still provide reasonable explanation about why urban population density can increase the PM_2.5_ pollution level. Existing studies suggesting a negative association between population density and air pollution concentration are mostly simulation studies with a focus on vehicular travel [27]. If we also consider the point-source air pollution, the relationship would be different. In this case, the point-source air pollution from pollution factories and building energy consumption (especially winter heating) are very likely to be positively correlated with urban population density, thus increasing PM_2.5_ concentration. This might help to further explain the fact some empirical studies also suggest a positive association between urban population density and air pollution level [33,57]. Another explanation would be transportation congestion effects associated with higher population density might outweigh the effects of transportation mode-shifting effects, especially in the context of urban China [39].

Our data do not support the finding that urban shape has significant influences on PM_2.5_ concentration, which is driven mostly by transportation air pollution emission, as is well documented in the previous literature [22,24,34,38,58]. Again, this can be explained by the fact that the PM_2.5_ Grids data we used in this study incorporate air pollutions from multiple sources including transportation and different point source pollution; thus, the effects of urban shape index (e.g., perimeter-to-area ratio) might not be significant.

As noted earlier, the finding that increased Heating Degree Days can lead to higher cardiorespiratory mortality ratio within the county conforms to some of the empirical research which reveals that heavily subsidized coal or indoor heating in winter months in China would bring about sustained exposure to particulate matter, which in turn reduce life expectancy [3]. Nevertheless, our data do not support the notion that there is a large indirect effect though increasing the PM_2.5_ concentration. Additionally, urban county can have direct impacts on cardiorespiratory mortality ratio, which is also confirmed with previous literature [27]. Since we have controlled several important urban form factors (e.g., population density, green space) and the number of pollution companies, it somehow makes sense that urban county itself does not have a significant influence on PM_2.5_ concentration, thus affecting the cardiorespiratory mortality ratio at the county level. GDP per capita does not show any significant impacts on PM_2.5_ concentration, which is somehow consistent with previous theories indicating the complexity of the relationship between GDP per capita and pollution level [59]. Previous studies based on major cities in China detected a negative relationship between GDP per capita and air pollution [24]. Since this study includes both urban and rural counties in China with a large variation in GDP level, the insignificant effects can be explained.

This study is subject to several limitations. First, because of data availability issues, our model does not include some important individual and household level characteristics (e.g., smoking habits, in-door air quality of the household, etc.), which might be the predominant cause of cardiorespiratory mortality. Second, the path model is built on the cross-sectional basis. Future research could consider constructing a longitudinal dataset to ensure a necessary time lag between air pollution exposure and mortality to better reveal causality. Third, this study uses cardiorespiratory mortality ratio at the county level as the only health outcome measure. Future research should explore the health impacts of the linkages in a more comprehensive way. For example, there has been study showing complex within-city tradeoffs in health outcomes associated with air pollution and physical activity. Urban form factor such as density could both affect residents’ exposure to air pollution and their intention for physical activity. Another relevant issue is the scale of our analysis: this study merely analyzed effects at the county level. Nevertheless, it was possible to analyze some more detailed factors governing relationships between urban form, air pollution, and health outcomes at a finer geographical scale. To sum up, more detailed research is needed to further identify various causal pathways.

## 6. Conclusions and Policy Implications

This study explored the causal pathways through which various urban form elements contribute to cardiorespiratory mortality by influencing PM_2.5_ concentration. We assembled a dataset by compiling a series of variables from disparate sources, including China’s Disease Surveillance Points (DSP) system, Chinese census, satellite Imagery, and the National Land Cover Database, and conducted a path analysis to quantify the indirect effects of urban form features on cardiorespiratory mortality based on a nationally representative sample of 158 counties in China. The results reveal that urban form elements, including population density, urban cohesion, and forest/green space ratio, have significant impacts on PM_2.5_ concentration, and are thus associated with the incidence of cardiorespiratory mortality at the county level. The results of this study also indicate that, compared with urban form elements, urbanization level of the county (“qu” vs. “xian”), percentage of elderly residents, and climate conditions (Heating Degree Days) are more predominant associating factors determining cardiorespiratory mortality rate at the county level.

This study offers a cautionary note about high population density, especially in the context of dense urban environments in China, may bring about negative health consequences related to air quality. Urban planners may consider using other relevant urban form strategies such as urban greening and ventilation path to relieve the possible negative effects. It is necessary to note that when incorporating lots of greenspace into a city, planners need to adopt careful spatial planning strategies to avoid the unintended consequences of bringing greater need to travel by car due to fragmentation. In this regard, transit-oriented developments with surrounded greenspace exemplified by Copenhagen’s finger plan would be a best practice case to follow [60]. Additionally, urban planners and policy makers are supposed to monitor the fragmentation level of rapid urbanizing areas on a regular basis, since fragmentary urban landscape contributes to increased urban pollution level and more cardiorespiratory mortality incidence. The study also reiterates that urban planning and design play an important role in promoting healthy cities. In this regard, a more comprehensive understanding of the complex relationships between the urban form features and public health would further guide urban planners and policy makers to the right policies and actions. More empirical work is also needed to evaluate specific urban form relevant policies and their causal pathways connecting health outcomes in various dimensions.

## Figures and Tables

**Table 1:** Definitions and sources of variables used in this study.

Variables	Definition	Source
Cardiorespiratory Mortality Rate	Cardiorespiratory mortality incidence per 100,000 population	China’s Disease Surveillance Points (DSP) system, which forms a nationally representative sample of mortality for the year 2005.
Annual PM_2.5_^1^	Population-weighted annual average PM_2.5_	Global Annual PM_2.5_ Grids from MODIS ^2^, MISR ^3^ and SeaWiFS ^4^ Aerosol Optical Depth (AOD) with GWR ^5^, v1 (2005); 1 km Grid Population Dataset of China
Population Density	Total population divided by total area within the county boundary	1 km Grid Population Dataset of China; County boundary shapefile of China
Urban Cohesion Index	Patch cohesion index measures the physical connectedness of the urban patch	National Land Use/Cover Database of China 2005 (30 m × 30 m); Calculated from GIS and Fragstats
Perimeter-Area Ratio	Area-weighted Perimeter-Area Ratio measures the shape complexity of urban landscape	National Land Use/Cover Database of China 2005 (30 m × 30 m); Calculated from GIS ^6^ and Fragstats
Forest/Green Space Ratio	Forest/green space area divided by total area within the county boundary	National Land Use/Cover Database of China 2005 (30 m × 30 m); Calculated from GIS and Fragstats
Heating Degree Days	Annual Heating Degree Days of the nearest temperature station	Calculated from 2005 daily temperature data From nationwide monitoring stations
Urban County	Dummy variable indicating if the county is urban or not	China Urban Statistical Yearbook 2005
Percentage of Elderly Residents (Age > 65)	Percentage of elderly residents with age >65	China Census Data 2000
Pollution Company Density	Total number of pollution companies divided by total area within the county boundary	Calculated from GIS; Nationwide Pollution Companies Data with geo information
GDP ^7^ Per Capita	GDP Per Capita	1 km Grid GDP dataset of China in 2005; County boundary shapefile of China

^1^ Atmospheric particulate matter with a diameter of less than 2.5 micrometers. ^2^ Moderate-resolution Imaging Spectroradiometer. ^3^ Multi-angle Imaging Spectroradiometer. ^4^ Sea-viewing Wide Field-of-view Sensor. ^5^ Geographically Weighted Regression. ^6^ Geographic Information System. ^7^ Gross Domestic Product.

**Table 2:** Descriptive statistics (N = 158).

Variables	M	SD	Min	Max
Cardiorespiratory Mortality Rate (per 10,000)	27.89	14.94	0.00	77.04
Annual PM_2.5_^1^ (μg/m^3^)	36.94	17.27	2.17	78.36
Population Density (/km^2^)	360.02	324.83	0.16	2132.77
Urban Cohesion Index	53.67	29.74	0.00	100.00
Perimeter-Area Ratio	28.33	9.08	0.00	40.00
Forest/Green Space Ratio (%)	28.37	26.52	0.00	89.96
Heating Degree Days	2530.61	1402.21	93.80	5893.20
Urban County	0.51	-	0.00	1.00
Percentage of Elderly Residents (Age > 65)	6.97	1.91	2.73	17.31
Pollution Company Density (/100 km^2^)	3.44	6.68	0.00	56.17
GDP ^2^ Per Capita (10,000 RMB ^3^)	4.50	10.49	0.02	89.44

M = mean; SD = standard deviation; Min = minimum value; Max = maximum value. ^1^ Atmospheric particulate matter with a diameter of less than 2.5 micrometers. ^2^ Gross Domestic Product. ^3^ RMB is abbreviation for Ren Min Bi, which is the official currency of China.

**Table 3:** The robust maximum likelihood estimates of unstandardized and standardized coefficient.

			Exogenous							
Endogenous	PopD	CohenI	PerimeR	ForestR	PollD	EldR	UrbanC	HDD	GDP	PM
PM										
β	0.021	−0.093	0.210	−0.091	3.637		−0.561	0.001	−0.075	
St. β	0.396	−0.162	0.11	−0.192	0.450		−0.016	0.116	−0.043	
z	3.07	−1.72	1.28	−2.63	4.81		−0.25	2.03	−0.69	
CardioR										
β						2.199	9.845	0.001	0.160	0.129
St. β						0.259	0.303	0.115	0.103	0.136
z						3.97	4.58	1.72	0.97	2.00

PopD: Population density; CohenI: Urban Cohesion Index; PerimeR: Perimeter-Area Ratio; ForestR: Forest/green space ratio; PollD: Pollution company density; EldR: Percentage of elderly residents; UrbanC: Urban county; HDD: Heating degree days; GDP: Gross domestic product per capita; PM: Population-weighted PM_2.5_; CardioR: Cardiorespiratory mortality ratio.
